# Towards the Optimization of Microwave-Assisted Extraction and the Assessment of Chemical Profile, Antioxidant and Antimicrobial Activity of Wine Lees Extracts

**DOI:** 10.3390/molecules27072189

**Published:** 2022-03-28

**Authors:** Dimitra Tagkouli, Thalia Tsiaka, Eftichia Kritsi, Marina Soković, Vassilia J. Sinanoglou, Dimitra Z. Lantzouraki, Panagiotis Zoumpoulakis

**Affiliations:** 1 Institute of Chemical Biology, National Hellenic Research Foundation, 48, Vas. Constantinou Ave., 11635 Athens, Greece; d_tagkouli@yahoo.gr (D.T.); tsiakath@uniwa.gr (T.T.); 2 Laboratory of Chemistry, Analysis & Design of Food Processes, Department of Food Science and Technology, University of West Attica, Ag. Spyridonos, 12243 Egaleo, Greece; ekritsi@uniwa.gr (E.K.); vsina@uniwa.gr (V.J.S.); 3 Institute for Biological Research “Siniša Stanković”—National Institute of Republic of Serbia, University of Belgrade, Bulevar Despota Stefana 142, 11000 Belgrade, Serbia; mris@ibiss.bg.ac.rs

**Keywords:** wine lees, microwave-assisted extraction (MAE), design of experiment (DoE), phenolic compounds, antiradical activity, infrared (IR) spectroscopy, antimicrobial activity

## Abstract

Wine lees, a sub-exploited byproduct of vinification, is considered a rich source of bioactive compounds, such as (poly)phenols, anthocyanins and tannins. Thus, the effective and rapid recovery of these biomolecules and the assessment of the bioactive properties of wine lees extracts is of utmost importance. Towards this direction, microwave-assisted extraction (MAE) factors (i.e., extraction time, microwave power and solvent/material ratio) were optimized using experimental design models in order to maximize the (poly)phenolic yield of the extracts. After optimizing the MAE process, the total phenolic content (TPC) as well as the antiradical, antioxidant and antimicrobial activity of the extracts were evaluated. Furthermore, Fourier transform infrared spectroscopy (FTIR) was employed to investigate the chemical profile of wine lees extracts. Red varieties exhibited higher biological activity than white varieties. The geographical origin and fermentation stage were also considered as critical factors. The white variety Moschofilero presented the highest antioxidant, antiradical and antimicrobial activity, while Merlot and Agiorgitiko samples showed noteworthy activities among red varieties. Moreover, IR spectra confirmed the presence of sugars, amino acids, organic acids and aromatic compounds. Thus, an efficient, rapid and eco-friendly process was proposed for further valorization of wine lees extracts.

## 1. Introduction

Discovering new end uses of agro-byproducts through the establishment of green and sustainable technologies for their valorization is a challenging task of pivotal importance [1]. Over the last years, the surge in the demand and consumption of wine (75% of the annual grape production) worldwide, and therefore the intensification of the winemaking process, has resulted in out-producing and discarding a huge volume of grape and wine byproducts (leaves, skin, stalk, stems, grape marc, seeds, wine lees, vine shoots, etc.) [1].

The byproduct accumulated at the bottom of vessels containing wine after the completion of alcoholic or malolactic fermentation, decanting, clarification, tartaric stabilization, filtration, storage, or other typical procedures of winemaking, as well as the residue recovered by filtration or centrifugation of this product, are referred to as wine lees [2,3]. Traditionally, wine lees are used during wine aging for improving the sensory attributes of wine, most importantly for the enhancement of the sensorial profile of wine and color stability [4].

Wine lees, although produced in lower quantities than the main winery byproduct, represent about 5% (*w*/*w*) of total grape mass used for vinification and an estimated annual residue for 2019 of about 0.88–2.66 million tones worldwide. This solid vinification byproduct presents a rich source of bioactive compounds [2,5], including tartaric acid, inorganic compounds, proteins, insoluble carbohydrates, phenolic compounds, yeast cells and other non-soluble residual grape material (seeds, skins. etc.). (Poly)phenolic compounds in wine lees are endowed with a high antiradical, antioxidant and metal chelating potential allowing them to act through biochemical mechanisms in order to delay oxidation phenomena preventing lipid oxidation and formation of peroxides [6,7]. The chemical profile of wine lees is highly depended on the *Vitis* variety, the agroclimatic conditions in vines, the geographical origin, oenological techniques and the time of wine aging [8,9]. The commercial potentials of wine byproducts and their rich-in-natural-compounds extracts are gaining attention. Wine lees and other wine residues can be used as (a) ingredients of novel functional foods [1,10], (b) fortification agents that furnish high nutritional value and stability to the final products, (c) antioxidant and antimicrobial factors, (d) industrial enzymes [11,12], (e) substrates for novel mushroom cultivars [13], (f) bioactive microencapsulated components [14] and biopolymers [2].

As the cornerstone in the pipeline of obtaining high-added value compounds and final products of excellent quality, the selection of an extraction technique of minimum environmental impact is of utmost significance [1]. The main pitfalls of conventional extraction approaches are extended extraction time, high required volumes of extraction solvents, low selectivity and, in certain cases, non-thermoprotective nature [15]. On the other hand, non-conventional extraction techniques (ultrasound-assisted extraction, pressurized liquid extraction, etc.) are engaging attention due to their short extraction time, reduced amounts of hazardous organic solvents and replacement with green innovative solvents (ionic liquids and deep eutectic solvents), maximization of extraction yields and reduced use of water and energy, lower risks and high reproducibility [15,16]. In addition, a large variety of bioactive components (i.e., (poly)phenols, carotenoids, polysaccharides, lignans, alkaloids, etc.) of different physicochemical properties (i.e., structure, polarity, volatility, etc.) are extracted from several matrices, among them wine and grape byproducts [1,17,18].

Considered a non-conventional extraction technique, microwave-assisted extraction (MAE) shares the advantages of the other non-traditional approaches. However, the two main drawbacks of this methodology are the poor extraction yields of volatile compounds due to the higher temperatures and the limited number of available extraction solvents, since a MAE solvent must absorb the microwave energy [15]. Apart from extraction solvent, other critical factors regarding MAE efficiency are extraction time, the ratio of solvent to material, microwave power and extraction temperature [15]. 

By apprehending the market dynamics and future perspectives of high-added value products, the aim of the present study was (a) to optimize the extraction process of bioactive compounds using MAE and experimental design and (b) to employ spectrophotometric, spectroscopic and microbiological analysis for the assessment of the biological activity, antimicrobial activity and chemical profile of the wine lees’ extracts. The overall goal was to evaluate the current findings, proposing an efficient and eco-friendly procedure for wine lees exploitation.

## 2. Results and Discussion

### 2.1. Extraction Solvent and Temperature

Among several extraction factors, the extraction solvent is a key variable for the fruitful upshot of MAE process. Normally, more polar solvents such as water or alcohols are used for MAE [19]. 

Acknowledging the fact that polyphenols are hydrophilic molecules, highly soluble in water–alcohols mixtures, three different polar solvent systems (ethanol, water and water:ethanol 1:1 *v*/*v*) were studied in order to obtain higher extraction yield (Table 1). Even though methanol is the most common solvent for the recovery of polyphenols, when MAE is implemented, ethanol is preferred. The polarity of the two alcohols is similar but ethanol is a better MWs absorber than methanol due to its higher ability to convert the microwave radiation to heat [20]. For the experiments, all extraction conditions were kept constant at certain values. In detail, 1 g of wine lees was dissolved in 20 mL of solvent. Extraction time was set at 10 min and MW power at 100 W. Extraction temperatures ranged between 80–100 °C in relation to the used solvent system.

The results indicate that the aqueous ethanol 1:1 *v*/*v* mixture provided a higher yield of TPC. Based on the current literature, binary solvent systems perform better in the MAE of polyphenols than mono-solvent systems, since the addition of water increases the polarity of the final extracting medium. This way, the extracting capacity of the solvent system is expanded from phenolic compounds of low polarity to phenolic compounds of high polarity. Moreover, a 1:1 mixture of water and ethanol delivered higher extraction yields due to the higher dielectric constant of water, which facilitates the absorption of thermal energy and the dissolution of target compounds [17,20].

In open-vessel MAE, the extraction temperature and MW power exhibit a mutualistic relationship since an increase in MW power always provokes an increase in the extraction temperature up to the boiling point of the extraction solvent. The raise of extraction temperature decreases solvent viscosity and accelerates the intracellular mass transfer of the analytes under study. Nonetheless, particular attention should be given in the excessive increase in extraction temperature, which may cause destruction of the structure of the investigated compounds with antioxidant activity [21].

In the present study, extraction temperature was adjusted to 85 °C. According to other published works, an MAE temperature around 80–85 °C increases the extraction yields of polyphenols compared with lower (30–40 °C) or higher (≥90 °C) temperatures [9,22].

### 2.2. Implementation of DOE Models for MAE Optimization

The extraction factors that were optimized using a 2^3^ full-factorial and Box–Behnken design were microwave (MW) power (W, X_1_), extraction time (min, X_2_) and solvent/material ratio (mL g^−1^, X_3_). Total (poly)phenols content (TPC) was used as the response factor in the DOE models. Ethanol–water 1:1 *v*/*v* mixture was the selected extraction solvent used for all experimental runs and extraction temperature was adjusted to 85 °C.

#### 2.2.1. Screening Design (2^3^ Full Factorial Design)

Normally, screening design is the first step of the optimization process necessary to direct the subsequent response surface design (Box–Behnken) in a more narrowed range of experimental values of the studied extraction factors where higher TPC was achieved. In order to prevent any systematic errors, the eight experiments of the 2^3^ full-factorial design were performed in random order. The results of the 2^3^ design are presented in Supplementary Materials Table S1. The suitability of the screening model was evaluated by the determination coefficient (R^2^) and the determination coefficient adjusted for the degrees of freedom (R^2^_adj_). The determination coefficient (R^2^) proves the goodness-of-fit of the dataset and the determination coefficient adjusted for the degrees of freedom (R^2^_adj_) establishes the equation terms, proposed by the models, which truly affect the response. A model where the values of R^2^ and R^2^_adj_ are higher than 0.8 and the difference between them is approximately equal to 0.2 interprets well the dataset. In regard to the present 2^3^ design, the direction provided by it could direct the BBD that follows in a value range where extraction factors should be maximized, since R^2^ = 0.843 and R^2^_adj_ = 0.725. The equation terms with *p*-values > 0.05 were excluded by the final 2^3^ design as non-significant terms. Based on the *p*-values of the model that were produced after the elimination of these terms, the final model pinpointed as most important factor for the construction of the BBD model the interaction of MW power and solvent/material ratio, x_1_x_3_ (*p*-value = 0.03) (Table S2a). The analysis of the two-dimensional (2D) contour plots revealed that the MAE of wine lees polyphenols was improved when MW power was adjusted at low values (Figure S1a–c).

#### 2.2.2. Response Surface Methodology (RSM)-Box–Behnken Design

Box–Behnken (BBD) design was implemented for the optimization of MAE process following the direction provided by the above-mentioned screening design. According to 2^3^ design, MW power should be examined at values between 40–60 W, while TPC appears to increase when the value range of extraction time and solvent/material ratio is extended to higher values (≥20 min and ≥50 mL g^−1^) than the ones implemented in the screening design. The experimental runs of BBD and (poly)phenols extraction yield, expressed in gallic acid equivalents (GAE) per one gram of dry sediment for each run, are demonstrated in Table S1. The processing of BBD results created a second-order polynomial equation for the determination of (poly)phenols extraction yield for wine lees samples (Equation (1)).

(1) Extraction yield (mg of GAE g^−1^ dry sediment) = 3.50 − 0.065 × x_1_ + 0.072 × x_1_^2^ + 0.21 × x_2_ + 0.16 × x_1_x_2_ + 0.10 × x_1_x_2_^2^ − 0.12 × x_1_^2^x_2_ − 0.29 × x_1_x_3_ − 0.080 × x_1_^2^x_3_

**Figure 1:**
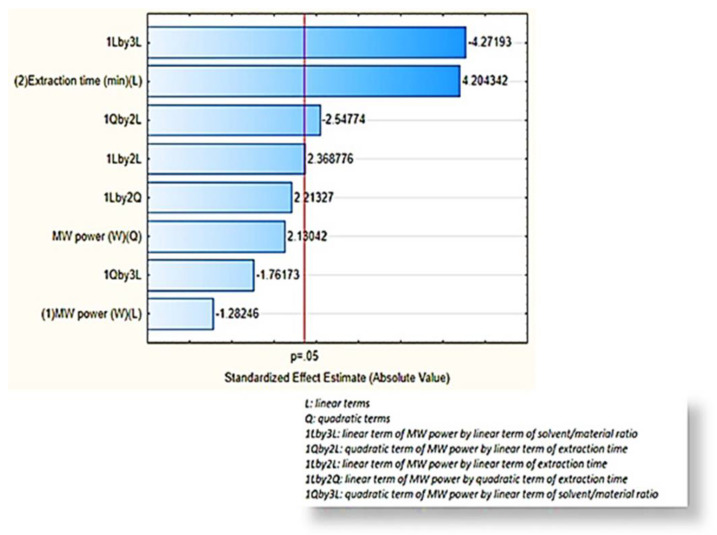
Pareto chart of the BBD model.

The order of significance of the equation terms is depicted in Pareto chart (Figure 1), where the most important terms are the ones that exceed the threshold of *p*-value ≤ 0.05 (red line). Based on the *p*-values presented in ANOVA table (Table S2b), (a) the interaction between the linear terms of MW power and the solvent/material ratio (x_1_x_3_), (b) the linear term of extraction time (x_2_), (c) the interaction between the quadratic term of MW power and the linear term of extraction time (x_1_^2^x_2_) and finally (d) the interaction between the linear term of MW power and the linear term of extraction time (x_1_x_2_) seems to affect essentially the final MAE extraction yield. The negative signs in two interaction terms, which involve MW power, highlighted that the increase in MW power leads to the decrease in extraction yield.

The reliability of the BBD model was ensured by the values of R^2^ and R^2^_adj_, which were high, and their difference was smaller than 0.2 (R^2^ = 0.889 and R^2^_adj_ = 0.762). According to the R^2^ value, almost 90% of the total variations was explained by the produced BBD model. Reckoning with the high *p*-value of the BBD model (*p*-value = 0.983) and not of each term individually, the experimental data fit well to the produced model and no lack-of-fit was observed. The robustness of the BBD model was assessed by the standard deviation of the four repetitions in the center points (0,0,0) (stdev = 0.20).

#### 2.2.3. Assessment of the Effects of the Extraction Factors under Optimization

**Figure 2:**
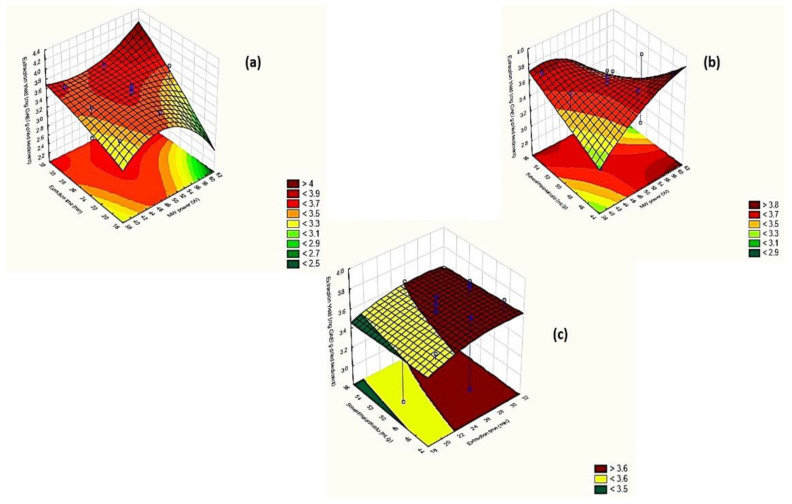
RSM plots of (**a**) extraction time vs MW power; (**b**) solvent/material ratio vs MW power; (**c**) solvent/material ratio vs extraction time.

The evaluation of the effect of extraction factors under optimization on the extraction yield was carried out by the generation of three-dimensional (3D) response surface methodology (RSM) plots (Figure 2a–c). RSM plots depict the interaction of two of the investigated extraction factors each time, while the third factor is always set at the medium value level (0).

##### MW Power

In most cases, higher values of MW power lead to higher extraction yields due to the more intense heating of extraction solvent and to the increase in extraction temperature for a short extraction duration [23]. The diffusion of polyphenols and their exudation from the substrate is easier and faster with higher MW power and consequently, higher temperatures are applied. Howbeit, the selectivity towards certain groups of bioactive groups is enhanced when lower MW power and longer extraction times are selected [20,24].

This assumption is also supported by Figure 2a, b, where relatively low MW power (50–60 W) achieved high polyphenols yields in longer extraction time (≥25 min) and solvent/material ratios (50–60 mL g^−1^).

##### Extraction Time

Despite the fact that the implementation of severe MW irradiation for a short time frame (from seconds to few minutes) can provide remarkable extraction yields, longer exposure of the samples at lower MW power improves the selective recovery of bioactive groups and protects them from thermal degradation [24].

According to Zhao et al. [25], the extraction efficiency of polyphenols is increased up to 45 min and then decreased. Based on Figure 2a,c, TPC of wine lees samples was maximized at extraction times over 25 min, when MW power was set at 50–60 W and solvent/material ratios at 50–60 mL g^−1^.

##### Solvent/material Ratio

Normally, higher solvent/material ratios induce higher concentration differences, which facilitate the analytes transfer and solubility in the extraction medium [26]. However, current findings presumed that after a critical solvent volume, the mass transfer reaches an equilibrium, thus a further increase in the extraction volume does not assist the extraction efficiency [25].

As it is evident from Figure 2b,c, maximum polyphenols yields were accomplished when solvent/material ratio was adjusted within the limits of 55–60 mL g^−1^.

##### Optimal Extraction Conditions of MAE

After the analysis of the BBD model, three experiments were conducted at the value region where TPC is maximized in order to select the optimal extraction conditions (Table S3). According to ANOVA analysis (*p*-value > 0.05), no significant statistical difference was observed between predicted and observed values of extraction yield, therefore, the produced BBD was able to predict reliably the (poly)phenols extraction yield. The final optimal extraction conditions are presented in Table 2.

One step further, MAE emerges as an easy, fast and competent extraction technique for the recovery of polyphenols compared with other extraction methodologies. The results of the present project are in line with the outcomes of other research works, where MAE provided higher total phenols content in significantly shorter times compared with conventional liquid extraction [4,6].

### 2.3. Analysis of Wine Lees Samples by Spectrophotometric Methods

The wine lees samples of different color, variety, geographical origin and fermentation stage were evaluated and classified in terms of their total phenolic content (TPC), antiradical (ABTS^●+^) and antioxidant (FRAP) activity on dry basis for the wine lees samples. The results are demonstrated in Table 3.

The total phenolic content (TPC) of wine lees varied from 3.57–26.0 mg of GAE g^−1^ dry wine lees in line with previously reported results [27]. The extract from red varieties (Samples 16–28) contained significantly higher amounts of (poly)phenols than the one from white varieties (Samples 1–15) due to the presence of (poly)phenolic compounds, such as tannins and anthocyanins [28].

Concerning the extracts of the white varieties, the Moschofilero variety (sample 15) was characterized by the highest concentrations in total polyphenols in accordance to Makris et al. [29]. Regarding the red varieties, sample 24 (Agiorgitiko variety) and 19 (Merlot variety), were found as the richer and poorer samples in polyphenols, respectively. As reported by Guendez et al. [30], Merlot wines had two-times lower polyphenol content compared with Agiorgitiko wines. Moreover, all Cabernet samples (21 and 22) and the majority of Agiorgitiko (23–26) contained high phenolic content in accordance with Rockenbach et al. [31], Lingua et al. [32] and Makris et al. [29].

Bearing in mind the groups that are framed based on the statistical differences between the samples (Table 3), other parameters that affect crucially the TPC of the samples were delineated. Besides the color of wine lees samples, factors such as grape variety, geographical origin or the fermentation stage [33] were considered as key indicators for the classification of wine lees samples.

The antiradical capacity of wine lees extracts ranged from 12.7–120.4 mg of TE g^−1^ dry sediment, which is in the range of the corresponding values reported by Rockenbach et al. [31] based on the variety color.

Following the trend of the TPC results, sample 15 (Moschofilero variety) presented the highest antiradical activity of white wine lees (Table 3) in accordance with similar studies results [34,35]. Among the red wine lees extracts, Sample 19 (Merlot variety) presented the lowest antiradical activity, while Sample 20 (Merlot variety) and 21 (Cabernet variety) the highest antiradical activity [36,37] (Table 3). Surprisingly, two samples of the same variety (Merlot variety) lied in the two opposites of value range, highlighting the importance of geographical region and fermentation stage, since Sample 19 was produced in a vineyard located in Attica, from a pre-fermented wine, whereas Sample 20 originated from Peloponnese and was acquired at a post-fermentation stage.

Ferric reducing power (FRAP) values extended from 15.9 to 243.8 mg of Fe (II) g^−1^ of dry sediment. Red varieties, irrespective the TPC and antiradical activity, presented higher antioxidant activity compared with white ones, which may be attributed to specific category of compounds such as anthocyanins [38].

From Table 3, Sample 1 (Kidonitsa variety) presented the lowest antioxidant activity among the samples of white varieties. Correspondingly, Samples 15 and 6 of Moschofilero and Savvatiano variety were those with a significant antioxidant activity. These results are in agreement with the results of Folin–Ciocalteu and ABTS^●+^ assay.

Regarding the antioxidant effectiveness of red varieties, the two Cabernet samples (Samples 21 and 22) were those with higher reducing power followed by an Agiorgitiko (Sample 26) and a Merlot (Sample 20) sample. All these samples were collected from a vineyard in Peloponnese in a post-fermentation stage, highlighting the significance of the vines’ location and oenological practices in the antioxidant activity of the wine lees.

Pearson’s correlation coefficients were calculated in order to review the degree of correlation between the three photometric methods applied.

High values of Pearson’s coefficients (R > 0.9) indicated an excellent positive correlation among TPC and antiradical activity (R = 0.952), TPC and antioxidant activity (R = 0.966) as well as between antiradical and antioxidant activity (R = 0.921). This denotes that wine lees’ antiradical and antioxidant capacity is tightly related to the phenolic groups of the extracts, establishing that the compounds of phenolic nature are those exhibiting antiradical and antioxidant properties. Furthermore, previous studies also verify the high correlation values of the photometric methods in wines and wine lees’ of both red and white varieties [28,39].

### 2.4. FT-IR Spectra Interpretation of Wine Lees Extracts

The Fourier Transform Infrared Spectroscopy (FT-IR) spectra of the wine lees extracts showed significant variations in the absorption regions and the absorption intensities. The interpretation of FT-IR spectra, in order to investigate the extracts chemical profile, were located among the wavenumbers from 3300 to 600 cm^−1^ and revealed the following results. All samples exhibited bands at the regions 3100–3050 and 1425–1380 cm^−1,^ which are assigned to stretching vibrations of C-H in aryl groups and of C-C in phenyl groups, respectively [40,41,42], as well as a band at 1350–1300 cm^−1^ corresponding to in-plane bending vibration of O-H of phenols [43]. This result proves the presence of (poly)phenolic compounds in wine lees extracts, in accordance to Folin–Ciocalteu’s assay results. Moreover, the absorbance bands at the region 1080–1020 cm^−1^ correspond to glucose, fructose and sucrose characteristic peaks [44,45]. According to the glucose, fructose and sucrose composition (Table 4), wine lees extracts were found highly heterogeneous in sugar moieties composition.

Furthermore, wine lees extracts gave bands in the regions 2940–2840, 1730–1700, 1600–1550, 1260–1200 and 950–900 cm^−1^, corresponding to C–H stretching of methyl- and methylene groups, to C=O stretching of organic acids, to the amino acids presence, to C–O stretching of glycerol and to C–C stretching of carbohydrates or flavonoids, respectively [46,47].

Interestingly, extracts of wine lees showed intensities in the region 880–850 cm^−1^. Para-substituted aromatic rings are characterized by one strong band near 850 cm^−1^ [40], therefore, sediments seem to retain the para-substituted aromatic compounds.

### 2.5. Antimicrobial Assay

Antibacterial potential of wine lees extracts towards bacteria of the American Type Culture Collection (ATCC) (Table 5) were presented in the following order, beginning with the extracts with the best antimicrobial potential (indicated with the lowest MIC values). All samples generally demonstrated lower minimum inhibitory (MIC) and minimum bactericidal concentration (MBC) values for ATCC bacteria compared with values for resistant strains. In general, the extracts of the red wines emerged as more effective antibacterial constituents compared with white wine extracts. This property is strongly related to the higher concentration of anthocyanins and tannins in red grapes [12]. The lowest range of observed MIC values (0.07–0.1 mg mL^−1^) was exhibited by the post-fermented red wines Merlot and Agiorgitiko, both originating from Peloponnese (Samples 20 and 25, respectively), while the highest MIC values (3.0–6.0/1.7–1.9 mg mL^−1^, respectively) were obtained for post-fermented white wine lees of the Kidonitsa and Savvatiano varieties (Samples 2 and 8, respectively).

As it is deduced from Table 5, post-fermented red wine lees of Merlot sample 20 from Peloponnese, which according to Table 3 displayed high TPC, exhibited higher antibacterial activity against Gram-negative bacteria, such as *E.coli* and *P. aeruginosa*, than against Gram-positive bacteria in accordance with other studies [48,49]. Instead, the other two Merlot wine lees (Sample 18 and Sample 19), which were produced in Attica before the wine fermentation, acted as antibacterial agents against both Gram-positive and Gram-negative strains, assigning the winery location and the fermentation stage as essential factors for the antibacterial activity of extracts.

Furthermore, Sample 25 of the Agiorgitiko variety showed significant inhibitory activity against the four Gram-positive and the four Gram-negative bacteria, as opposed to all the other Agiorgitiko samples regardless of the vineyard and processing. Recent studies [50,51] reported that the effect of additional viticultural and oenological variables, such as the heterogeneity of the type of the vines/grapes used for vinification, field management, soil composition, maceration conditions and weather conditions at which the vines are exposed, is related to the bactericidal effect of wine lees.

According to Table 5, *B. cereus* was the most sensitive strain to treatment with wine lees extracts among all of the strains tested in this study, since fifteen out of twenty-eight wine lees samples showed, on average, lower MIC values against *B. cereus* compared with other bacteria, followed by *S. aureus*. On the contrary, *E. coli* is proven to be the most resistant strain to antimicrobial treatment with wine lees (Table 5).

## 3. Materials and Methods

### 3.1. Reagents and Standards

Folin–Ciocalteu’s (FC) phenol reagent was supplied from Merck KGaA (Darmstadt, Germany). Gallic acid (3,4,5-trihydroxybenzoic acid) was obtained from Alfa Aesar GmbH&Co (Karlsruhe, Germany). Trolox (6-Hydroxy-2,5,7,8-tetramethylchroman-2-carboxylic acid) and potassium persulfate were purchased from Sigma-Aldrich (St. Louis, MO, USA). 2,2′-Azino-bis (3-ethylbenzothiazoline-6-sulfonic acid ammonium salt) (ABTS^●+^) was purchased from Tokyo Chemical Industry Co. LTD (Tokyo, Japan). 2,4,6-tris(2-pyridyl)-S-triazine (TPTZ) and iron (III) chloride hexahydrate were obtained from Sigma Chemical (St. Louis, MO, USA). All reagents used were of analytical grade and were purchased from Sigma-Aldrich (Saint Quentin Fallavier, France), Merck KGaA (Darmstadt, Germany) and Carlo Erba Reagents (Val de Reuil, France).

### 3.2. Wine Sees Sample-Set and Sample Preparation

Wine lees samples (N = 28) of white and red grape varieties were collected during the vinification period in autumn 2016 from different wineries from the Attica and Peloponnese regions in Greece. Samples with their general characteristics are presented in Table 6. Until further treatment, samples were kept in glass bottles in −20 °C in darkness.

**Figure 3:**
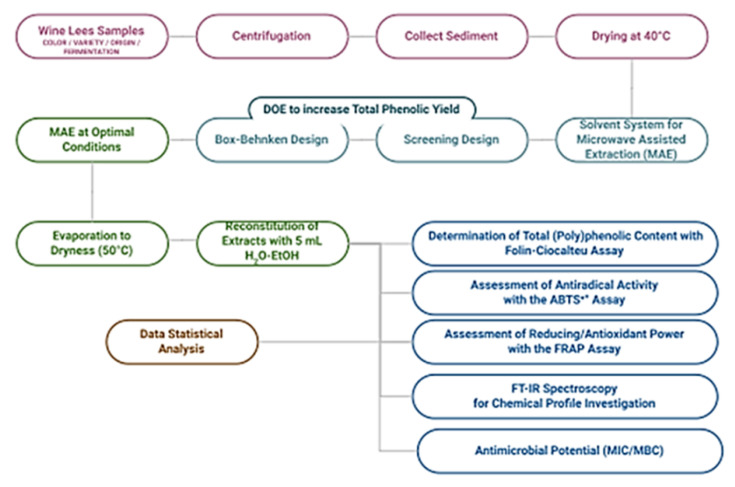
Flowchart of experimental process.

After sample collection, all samples were centrifuged at 2100 G for 10 min at 10 °C in order to separate the wine lees’ sediment from the supernatant, which was discarded. The sediment was then dried in an oven at 40 °C until reaching a stable weight to remove sample moisture and to suspend any enzymatic and microbial activity. Dried material was homogenized and powdered in a laboratory mill (Type ZM1, Retsch GmbH, Haan, Germany). Dry material and samples were placed in glass jars and vials at −20 °C until further process and analysis. The moisture of wine lees sediments varied from 40–80% as shown in Table S4. The flowchart of the developed experimental platform is presented in Figure 3.

### 3.3. Microwave-Assisted Extraction (MAE) Instrumentation and Process

Microwave-assisted extraction (MAE) was performed using a CEM Focused Microwave Synthesis System, Model Discover (CEM Corporation, Matthews, NC, USA) in open-vessel mode with a reflux system adjusted over the open cell.

Ethanol, water and a 1:1 *v*/*v* mixture of ethanol:water were the extraction solvents used. One gram (1 g) of dried wine lees sediment was used for the extraction. The solvent volume was not the same in all experimental runs since solvent-to-material ratio was one of the factors under optimization of the experimental design models. After MAE, the extract was filtered with Whatman paper filter and evaporated to dryness by rotary evaporation at 50 °C. Then, the dry residue of the extract was collected with 5 mL of the extraction solvent. Aliquots of the extracts were used for the (a) spectrophotometric, (b) IR and (c) antimicrobial analyses.

### 3.4. Experimental Design (DOE) Models

The screening and the optimization of the extraction factors were carried out on the dry residue of the wine lees sediment by implementing a two-level full factorial design, 2^3^, and a symmetrical 16-run three-level Box–Behnken design (BBD), respectively. The examined extraction factors were the (a) microwave (MW) power, X_1_ (W); (b) extraction time, X_2_ (min) and (c) solvent/material ratio, X_3_ (mL g^−1^). The extraction yield of (poly)phenols, expressed as mg of gallic acid equivalents (GAE) per gram of dry sediment, was chosen as the measured response of the two DOE models.

In order to settle on the optimal extraction conditions that allow the recovery of (poly)phenols from samples of both high and low concentration, the wine lees sample used for the DOE process belonged to the white variety Savvatiano (Sample 7, Table 4), since white varieties contain lower concentrations of (poly)phenols than red varieties [28]. In addition, the Savvatiano variety is the most representative variety of the current sample set as it includes the majority of white wine samples.

In order to assure that DOE models provide unbiased results, the real values of the extraction variables, which are expressed in different physical units (i.e., Watt, minutes, volume-to-weight, etc.), were transformed to coded normalized dimensionless values (x_1_, x_2_, x_3_) [52]. Real and normalized values of the extraction factors for the two DOE models are presented in Table S5.

### 3.5. Spectrophotometric Analyses

#### 3.5.1. Total Phenolic Content (TPC)

The total phenolic content (TPC) of each sample was determined by applying a micromethod of Folin–Ciocalteu’s (FC) colorimetric assay [53]. The results were expressed as mg of gallic acid equivalents (GAE) per 1 g of dried sediment, using a standard curve with a range of 25–500 mg L^−1^ gallic acid (y = 0.001 × x + 0.003, R^2^ = 0.997). The photometric measurements were performed at 750 nm.

#### 3.5.2. Scavenging Activity on 2,2′-Azino-bis-(3-ethylbenzothiazoline-6-sulfonic acid) Radical (ABTS^●+^)

For each sample, the antiradical activity was determined as described in previous works [53]. Trolox, a water-soluble form of vitamin E, was used as a standard compound, and the antiradical activity of each sample was expressed as mg of Trolox equivalents (TE) per 1 g of dried sediment, using a standard curve with a concentration range of 0.20–1.5 mM (y = 0.282 × x − 0.002, R^2^ = 0.995). Sample measurements were conducted at 734 nm.

#### 3.5.3. Ferric Reducing/Antioxidant Power Assay (FRAP)

The Ferric reducing/antioxidant power for each sample was evaluated based on the reduction in Fe(III) in the form of ferric-2, 4,6-tripyridyl-s-triazine complex to Fe(II), as described by Lantzouraki et al. [53]. A standard curve was prepared using various concentrations (600–2000 μM) of FeSO_4_·7H_2_O stock solutions. The results are expressed as mg of Fe (II) per 1g of dried sediment (y = 0.00027 × x − 0.011, R^2^ = 0.997). The analysis was carried out at 593 nm.

### 3.6. Fourier Transform Infrared Spectroscopy (FTIR)

FT-IR spectra were collected with an Alpha-P spectrometer (Bruker, Billerica, MA, USA), the Alpha FT-IR wine analyzer (Bruker Optics) on a diamond ATR crystal covered with a flow through cell, facilitating sample injection. The Alpha-P instrument has a potassium bromide (KBr) beam splitter and a 2 × 2 mm temperature controllable ATR diamond crystal sample plate, which was set at 40 °C. The instrument was fitted with OPUS software (OPUS version 7.2 for Microsoft Windows, Bruker Optics, Billerica, MA, USA). No further sample preparation was performed for spectral analysis and volumes of 5 mL were used. The spectrum of each sample and background were obtained from 4000 to 375 cm^−1^ and the average of 64 scans at a resolution of 8 cm^−1^ with a scanner velocity of 7.5 kHz was recorded. One background measurement was taken before each sample measurement. The ALPHA Wine Analyzer comes with a starter calibration that was assembled by the accredited (DAkkS) Institute Heidger (Kesten, Germany) and contains more than 1700 wines from wine producing countries worldwide. The organic acid and sugar contents were measured for each wine lees sample using the “ALPHA wine analyzer” apparatus and the starter calibration curves dedicated to each of the determined compounds.

### 3.7. Antimicrobial Activity

#### 3.7.1. Tested Microorganisms

Four Gram (+) bacteria (*Bacillus cereus* clinical isolate, *Micrococcus flavus* ATCC 10240, *Staphylococcus aureus* ATCC 6538 and *Listeria monocytogenes* NCTC 7973), four Gram (−) bacteria (*Escherichia coli* ATCC 35210, *Enterobacter cloacae* human isolate, *Pseudomonas aeruginosa* ATCC 27853 and *Salmonella typhimurium* ATCC 13311) and three resistant bacteria, Methicillin-resistant *Staphylococcus aureus* (MRSA), *Escherichia coli* and *Pseudomonas aeruginosa* PAO1, were used for testing of antibacterial activity of wine less products. The microorganisms are deposited in the Mycological Laboratory, Department of Plant Physiology, Institute for Biological Research “Siniša Stanković”, University of Belgrade, Belgrade, Serbia. The bacterial suspensions were adjusted with sterile saline to a concentration of 1.0 × 10^5^ CFU mL^−1^. The inocula were prepared daily and stored at 4 °C until use. Dilutions of the inocula were cultured on solid medium to verify the absence of contamination and to check the validity of the inoculum. All experiments were performed in duplicate and repeated three times. The isolation and determination of clinical bacteria is described in Supplementary Materials [54,55].

#### 3.7.2. In Vitro Assays for Determination of Antibacterial Activity—Microdilution Method

The compounds were tested on antibacterial activity using a microdilution method [55,56]. Minimal inhibitory (MIC) and minimal bactericidal (MBC) concentrations of the tested samples were determined by serial dilutions of compounds dissolved in 5% DMSO-water solution in 96-well microtitre plates. Bacterial inoculum, 1.0 × 10^4^ CFU mL^−1^, was added to LB medium and compounds dissolved in 5% DMSO solution containing 0.1% Tween 80 (*v*/*v*) (1 mg mL^−1^). Minimal inhibitory concentration (MIC) was defined as the lowest concentration that inhibited bacterial growth, without visible growth, at the binocular microscope. The lowest concentration with no visible growth was defined as the MBC, indicating 99.5% killing of the original inoculum. All wells were measured at a wavelength of 655 nm by Microplate manager 4.0 (Bio-Rad Laboratories, Hercules, California, USA) and compared with a blank and the positive control. Antibiotics used as a positive control were Streptomycin (Sigma P 7794) and Ampicillin (Panfarma, Belgrade, Serbia) (1 mg mL^−1^ in sterile physiological saline), while 5% DMSO was used as a negative control. All experiments were performed in duplicate and repeated three times.

### 3.8. Data Analysis

Data processing was conducted and graphs were produced using the Statistica package (Version 12, TIBCO Software Inc., Palo Alto, CA, USA). All measurements were realized at 95% (*p*-values ≤ 0.05) confidence level. Normal or non-normal distribution of the samples was confirmed by using the Shapiro–Wilks test. All data were normally distributed with the exception of BBD model data. However, since the number of samples/observations was larger than 30, normality can be assumed for the BBD dataset [57]. In all cases, the pairwise multiple comparison of the samples’ averages to determine whether there is any statistical difference between them and which of them differ significantly was performed by one-way ANOVA and Tukey honestly significant difference post hoc analysis. The correlation of spectrophotometric results was carried out using Pearson’s correlation.

## 4. Conclusions

Wine lees are the byproduct accumulated at the bottom of vessels containing wine after the completion of winemaking processes, as well as the residue recovered by filtration or centrifugation of this byproduct [2]. As the cornerstone in the pipeline of obtaining high-added value final products of excellent quality and beneficial biological activities, the selection of an extraction technique of reduced environmental impact is of utmost significance [1].

In the present study, MAE process was optimized using two-level full-factorial and Box–Behnken design in order to achieve higher TPC values. Ethanol and water mixture at a 1:1 ratio was selected as the best extraction solvent, while extraction temperature was set at 85 °C. Extraction time at 35 min, MW power at 54 W and solvent/material ratio at 60 mL g^−1^ were determined as the optimal values of extraction factors. In general, white varieties showed lower FRAP and ABTS^•+^ values than red varieties, which contained higher amounts of (poly)phenols, tannins and anthocyanins.

The same trend was also presented in the antimicrobial assays, where red varieties exhibited strong antimicrobial activities especially against *B. aureus* strains. Based on the results, samples of the white variety Moschofilero were the ones with the most significant biological activities. On the other hand, samples of Merlot and Agiorgitiko red varieties demonstrated the most satisfactory antioxidant, antiradical and antimicrobial activity. However, factors other than grape variety, such as the vineyard location and the fermentation stage, also emerged as crucial factors affecting wine lees biological activities. Post-fermented red wine lees samples from Peloponnese showed higher TPC, ABTS^•+^ and FRAP values and lower MIC values against Gram-negative bacteria. On the other hand, red wine lees extracts obtained at pre-fermentation stage from Attica displayed high antibacterial activity against both Gram-negative and Gram-positive bacteria. Furthermore, the interpretation of FT-IR bands highlighted the presence of sugars (glucose, fructose and sucrose), amino acids, organic acids and para-substituted aromatic compounds in wine lees extracts.

By overviewing the results of the present study, MAE could emerge as a suitable alternative for the recovery of extracts with strong antioxidant and antimicrobial activity from winemaking by-products by replacing the laborious and time-consuming conventional extraction techniques.

## Figures and Tables

**Table 1:** Extraction yields of the three investigated solvent systems expressed as total phenolic content (mg GAE g^−1^ dry sediment).

Extraction Solvent (*v*/*v*)	Extraction Yield (mg of GAE g^−1^ Dry Sediment) (± stdev), *n* = 3 ^1^
Ethanol	1.60 (± 0.21) ^b^
Water	0.46 (± 0.21) ^c^
Ethanol:water 1:1	2.034 (± 0.064) ^a^

^1^ *n* = the number of replicates; ^a,b,c^: values with different lowercase letters are significantly different (*p*-value ≤ 0.05).

**Table 2:** Optimal MAE conditions.

Extraction Factors	Optimal Conditions
MW power (W)	54
Extraction time (minutes)	35
Solvent/material ratio (mL g^−1^)	60
Extraction yield (mg of GAE g^−1^ dry sediment) (±stdev), *n* = 3 ^1^	3.58 (±0.17)

^1^ *n* = the number of measurements replicates.

**Table 3:** Total phenolic content, antiradical activity by ABTS^•+^ method and antioxidant activity by FRAP method for wine lees extracts.

A/A. ^2^	Average mg of GAE g^−1^ of Dry Sediment (±S.D.), *n* = 3 ^1^	Average mg of TE g^−1^ of Dry Sediment (±stdev), *n* = 3 ^1^	Average mg of Fe (II) g^−1^ of Dry Sediment (±stdev), *n* = 3 ^1^
1	3.57(±0.40) ^k^	33.7(±1.8) ^i,j^	15.9(±1.9) ^r^
2	5.81(±0.69) ^h,i,j,k^	20.7(±2.7) ^l,m^	46.7(±3.7) ^m,n,o^
3	4.82(±0.95) ^i,j,k^	15.77(±0.80) ^m,n^	52.86(±0.58) ^l,m^
4	4.28(±0.31) ^j,k^	12.67(±0.90) ^n^	35.4(±1.4) ^p,q^
5	7.65(±0.84) ^g,h,i,j^	40.6(±2.9) ^g,h,i^	76.1(±5.2) ^k^
6	11.5(±2.1) ^e,f,g^	48.4(±1.3) ^f^	103.8(±5.0) ^i^
7	7.48(±0.88) ^h,i,j,k^	48.2(±2.3) ^f^	44.7(±2.1) ^m,n,o,p^
8	4.09(±0.65) ^j,k^	30.7(±1.4) ^j,k^	32.7(±2.1) ^q^
9	5.61(±0.72) ^h,i,j,k^	20.6(±3.0) ^l,m^	57.7(±1.9) ^l^
10	8.72(±0.81) ^f,g,h,i^	24.6(±4.0) ^k,l^	76.0(±3.9) ^k^
11	5.1(±1.3) ^i,j,k^	25.4(±1.3) ^k,l^	34.43(±0.39) ^q^
12	6.9(±1.2) ^h,i,j,k^	40.8(±1.5) ^g,h,i^	39.5(±2.7) ^o,p,q^
13	6.01(±0.25) ^h,i,j,k^	43.3(±2.4) ^f,g,h^	41.63(±0.58) ^n,^^o,p,q^
14	6.73(±0.84) ^h,i,j,k^	42.41(±0.90) ^f,g,h^	49.5(±2.7) ^l,m,n^
15	13.22(±0.79) ^d,e^	67.3(±3.8) ^e^	117.5(±1.7) ^h^
16	17.2(±1.2) ^c^	72.2(±2.3) ^d,e^	151.1(±2.3) ^f^
17	16.9(±2.0) ^c,d^	76.5(±3.5) ^d^	178.9(±3.1) ^e^
18	12.6(±1.6) ^e,f^	36.4(±1.7) ^h,i,j^	89.3(±0.97) ^j^
19	7.10(±0.85) ^h,i,j,k^	25.2(±2.5) ^k,l^	58.85(±0.39) ^l^
20	22.7(±1.2) ^a,b^	120.4(±2.2) ^a^	224.8(±6.4) ^b^
21	25.3(±1.5) ^a^	120.4(±3.6) ^a^	243.8(±1.4) ^a^
22	24.0(±2.8) ^a^	113.5(±3.5) ^a,b^	234.2(±3.7) ^a,^^b^
23	24.2(±1.5) ^a^	100.9(±2.5) ^c^	192.5(±1.5) ^d^
24	26.0(±1.1) ^a^	114.08(±0.30) ^a,b^	203.4(±4.6) ^c^
25	18.6(±1.1) ^c^	111.5(±0.70) ^b^	139.8(±3.1) ^g^
26	19.0(±1.6) ^b,c^	94.2(±1.2) ^c^	231.4(±3.7) ^b^
27	8.0(±1.4) ^g,h,i,j^	43.9(±4.0) ^f,g^	70.3(±1.7) ^k^
28	9.25(±0.63) ^f,g,h^	41.4(±1.9) ^f,g,h^	78.0(±5.6) ^k^

^1^ *n* = the number of measurements replicates; ^a–r:^ values with different lowercase letters in the same column are significantly different (*p*-value ≤ 0.05). ^2^ The samples details are presented in Table 6 (Section 3).

**Table 4:** Organic acid and sugar content of the sediment extracts of wine lees expressed as mg g^−1^ of dry sample.

A/A	Citric Acid	Lactic Acid	Tartaric Acid	Glycerol	Fructose	Glucose	Saccharose
1	N.D.	5.9	15.5	57.0	131.5	213.9	3.0
2	6.9	9.0	17.2	53.5	1.5	N.D.	8.5
3	4.4	4.5	15.3	50.5	22.5	12.5	3.0
4	7.7	9.1	4.1	57.5	12.0	14.5	N.D.
5	N.D.	5.3	16.5	60.5	193.9	113.5	N.D.
6	4.2	9.6	14.8	73.4	167.4	71.9	1.0
7	6.5	17.2	16.8	52.5	N.D.	N.D.	8.5
8	11.5	11.8	16.0	45.0	N.D.	N.D.	6.0
9	5.8	5.1	18.2	32.0	2.0	4.0	11.0
10	16.0	8.0	49.9	81.6	N.D.	8.3	21.6
11	15.9	14.3	0.1	43.0	N.D.	10.5	0.5
12	4.3	9.5	18.2	35.5	4.5	3.5	6.5
13	3.4	10.7	19.4	43.0	6.5	3.0	8.0
14	8.1	9.2	17.0	41.5	1.5	7.0	10.5
15	9.3	13.1	14.3	68.0	11.5	6.5	7.0
16	3.7	6.8	20.2	66.0	6.0	11.0	4.0
17	4.2	9.7	18.1	62.9	12.5	8.0	9.5
18	N.D.	4.8	17.3	61.5	192.5	141.0	N.D.
19	N.D.	2.0	20.5	56.0	178.9	134.4	N.D.
20	6.8	8.9	17.0	56.0	3.5	1.0	8.0
21	6.7	8.2	14.6	63.0	1.0	4.5	3.5
22	5.4	9.6	15.4	70.0	3.5	3.5	6.5
23	9.0	7.9	11.1	44.5	N.D.	3.0	13.0
24	7.5	11.4	15.9	66.0	5.5	6.0	4.5
25	3.8	7.3	14.3	50.0	1.0	6.0	5.0
26	5.3	7.9	22.9	61.5	6.0	11.5	10.0
27	0.2	16.2	24.4	74.5	13.0	N.D.	5.0
28	4.7	13.8	17.3	54.0	8.0	3.0	8.0

**Table 5:** Antibacterial activity of wine lees sediment against ATCC bacteria (mg mL^−1^).

A/A	MIC/MBC Values	*E.c.*	*S.t.*	*En.cl.*	*P.a*	*L.m*	*M.f.*	*B.c.*	*S.a.*
1	MIC	1.13 ± 0.11 ^a,b,k^	0.63 ± 0.06 ^a,b,c,g^	0.67 ± 0.11 ^a,b^	0.6 ± 0.00 ^a,b,c,d^	0.53 ± 0.11 ^a,b,d^	0.56 ± 0.06 ^a,b,c,e^	0.67 ± 0.11 ^a,b,c,e^	0.63 ± 0.06 ^a,b,c,e^
	MBC	2.27 ± 0.23 ^a,b,f,i^	1.30 ± 0.17 ^a,f^	1.13 ± 0.11 ^a,b^	1.2 ± 0.00 ^a,b,j,e^	1.10 ± 0.17 ^a,e^	1.30 ± 0.17 ^a,b,c,d^	1.13 ± 0.11 ^a,b,f^	1.23 ± 0.06 ^a,d^
2	MIC	6.63 ± 0.11 ^a^	3.40 ± 0.09 ^a^	3.35 ± 0.00 ^a^	3.23 ± 0.2 ^a^	3.30 ± 0.1 ^a^	3.27 ± 0.14 ^a^	6.47 ± 0.4 ^a^	3.23 ± 0.2 ^a^
	MBC	13.33 ± 0.3 ^a^	6.47 ± 0.4 ^a^	6.63 ± 0.11 ^a^	6.7 ± 0.00 ^a,b^	6.57 ± 0.23 ^a^	6.47 ± 0.4 ^a^	13.33 ± 0.3 ^a^	6.47 ± 0.4 ^a^
3	MIC	0.3 ± 0.00 ^a,h^	0.53 ± 0.11^a,c^	0.33 ± 0.06 ^a,b,d,e^	0.6 ± 0.00 ^a,b,c^	0.67 ± 0.11 ^a,b,c,e^	0.27 ± 0.06 ^a,b^	0.37 ± 0.11 ^a^	0.33 ± 0.06 ^a^
	MBC	0.67 ± 0.11 ^a,b^	1.13 ± 0.11 ^a^	0.73 ± 0.23 ^a,b,d^	1.13 ± 0.11 ^a,b,e^	1.2 ± 0.00 ^a,b,c,e^	0.53 ± 0.11 ^a,c,d^	0.6 ± 0.00 ^a,b^	0.67 ± 0.11 ^a,b^
4	MIC	3.23 ± 0.11 ^a^	1.6 ± 0.00 ^a,b^	1.53 ± 0.11 ^a^	1.63 ± 0.06 ^a,b^	1.67 ± 0.11 ^a^	3.20 ± 0.17 ^a^	0.87 ± 0.11 ^a^	3.20 ± 0.17 ^a^
	MBC	6.40 ± 0.35 ^a^	3.7 ± 0.23 ^a^	3.20 ± 0.17 ^a^	3.37 ± 0.11 ^a^	3.3 ± 0.00 ^a,b^	6.40 ± 0.35 ^a^	1.53 ± 0.11 ^a,b,c,f^	6.57 ± 0.06 ^a,b^
5	MIC	0.93 ± 0.06 ^a,b^	0.37 ± 0.14 ^a,e^	0.53 ± 0.14 ^a,b^	0.42 ± 0.06 ^a,f^	0.47 ± 0.03 ^a,b,d^	0.50 ± 0.09 ^a,b,f^	0.45 ± 0.00 ^a^	0.47 ± 0.03 ^a,e^
	MBC	1.87 ± 0.11 ^a,b,i^	0.9 ± 0.11 ^a,b,f,g^	0.83 ± 0.11 ^a,b^	0.77 ± 0.23 ^a,b^	0.83 ± 0.06 ^a,b,c,g^	0.93 ± 0.06 ^a,b,c^	0.83 ± 0.11 ^a,b,f^	0.93 ± 0.06 ^a,b,c^
6	MIC	0.87 ± 0.11 ^a,b^	0.93 ± 0.23 ^a,b,c,e,g^	0.83 ± 0.06 ^a,b,d^	0.8 ± 0.00 ^a,b,c,i^	0.7 ± 0.17 ^a,b^	0.73 ± 0.11 ^a^	0.33 ± 0.11 ^a^	0.73 ± 0.11 ^a,c,g^
	MBC	1.57 ± 0.06 ^a,b,i^	1.6 ± 0.00 ^a,b,f^	1.73 ± 0.23 ^a,d^	1.53 ± 0.11 ^a,b^	1.56 ± 0.06 ^a^	1.6 ± 0.00 ^a,b,c,d^	0.87 ± 0.11 ^a,b,c,f^	1.47 ± 0.23 ^a,e^
7	MIC	0.93 ± 0.06 ^a,b^	0.47 ± 0.03 ^a^	0.5 ± 0.1 ^a,b,d^	0.45 ± 0.00 ^a,d^	0.40 ± 0.1 ^a,f^	0.43 ± 0.03 ^a,f,e^	0.45 ± 0.00 ^a^	0.48 ± 0.06 ^a^
	MBC	1.73 ± 0.11 ^a,i^	0.93 ± 0.06 ^a,b,d^	0.87 ± 0.06 ^a,b^	0.93 ± 0.06 ^a,b,j^	0.77 ± 0.23 ^a,b,c^	0.97 ± 0.11 ^a,b,c^	0.9 ± 0.00 ^a,b,d^	0.93 ± 0.06 ^a,b,c^
8	MIC	1.9 ± 0.08 ^a,b^	1.73 ± 0.2 ^a^	0.95 ± ±0.04 ^a^	1.85 ± 0.00 ^a,b^	1.77 ± 0.14 ^a,b^	1.85 ± 0.00 ^a^	1.90 ± 0.09 ^a^	0.95 ± 0.04 ^a,b,e,i^
	MBC	3.7 ± 0.00 ^a^	3.63 ± 0.11^a^	1.90 ± 0.09 ^a,d^	3.47 ± 0.4 ^a^	3.63 ± 0.11 ^a^	3.7 ± 0.00 ^a^	3.63 ± 0.11 ^a^	1.85 ± 0.00 ^a,b,d^
9	MIC	2.53 ± 0.5 ^a^	2.7 ± 0.17 ^a^	1.43 ± 0.06 ^a^	1.27 ± 0.2 ^a,b^	2.8 ± 0.00 ^a^	2.73 ± 0.11 ^a^	0.38 ± 0.01 ^a^	1.33 ± 0.11 ^a^
	MBC	5.80 ± 0.17 ^a^	5.6 ± 0.11 ^a^	2.87 ± 0.11 ^a^	2.70 ± 0.17 ^a^	5.47 ± 0.4 ^a^	5.63 ± 0.11 ^a^	0.83 ± 0.15 ^a,b^	2.53 ± 0.46 ^a,b^
10	MIC	0.5 ± 0.00 ^a,b^	0.15 ± 0.04 ^a,g^	0.27 ± 0.02 ^a,b^	0.14 ± 0.01 ^a,b^	0.15 ± 0.04 ^a,b^	0.27 ± 0.02 ^a,b^	0.17 ± 0.07 ^a,b,c^	0.12 ± 0.17 ^a,c,k^
	MBC	1 ± 0.00 ^a,b,c,f,i^	0.34 ± 0.11 ^a,b,g^	0.58 ± 0.10 ^a,b^	0.32 ± 0.1 ^a^	0.34 ± 0.13 ^a,c,f^	0.68 ± 0.27 ^a,b,c^	0.26 ± 0.00 ^a,b^	0.29 ± 0.05 ^a,c^
11	MIC	0.68 ± 0.01 ^a,b,j^	1.4 ± 0.09 ^a^	0.72 ± 0.07 ^a,b,c^	1.35 ± 0.00 ^a,b^	1.40 ± 0.1 ^a,b^	0.70 ± 0.04 ^a,b,e^	0.67 ± 0.00 ^a,b,d^	1.40 ± 0.09 ^a,i^
	MBC	1.23 ± 0.20 ^a,b^	2.47 ± 0.4 ^a^	1.40 ± 0.08 ^a,b,d^	2.63 ± 0.11 ^a^	2.7 ± 0.00 ^a,b^	1.30 ± 0.09 ^a,b^	1.23 ± 0.2 ^a,b^	2.63 ± 0.11 ^a^
12	MIC	0.33 ± 0.06 ^a,b,k^	0.53 ± 0.11 ^a^	0.37 ± 0.11 ^a,b,c^	0.67 ± 0.11 ^a,b^	0.63 ± 0.06 ^a,e^	0.7 ± 0.06 ^a,b^	0.23 ± 0.11 ^a,e^	0.27 ± 0.06 ^a,g^
	MBC	0.73 ± 0.23 ^a,b^	1.13 ± 0.11 ^a,c^	0.6 ± 0.00 ^a,b^	1.2 ± 0.00 ^a,b,e^	1.10 ± 0.17 ^a^	0.70 ± 0.17 ^a,d^	0.6 ± 0.00 ^a,b^	0.73 ± 0.23 ^a,e^
13	MIC	0.53 ± 0.06 ^a,b,d^	0.33 ± 0.14 ^a,c^	0.25 ± 0.00 ^a^	0.33 ± 0.14 ^a,b,d^	0.32 ± 0.11 ^a,b^	0.25 ± 0.00 ^a^	0.27 ± 0.03 ^a^	0.31 ± 0.11 ^a^
	MBC	1 ± 0.00 ^a,b,i^	0.67 ± 0.3 ^a,f^	0.60 ± 0.17 ^a,b^	0.5 ± 0.00 ^a^	0.57 ± 0.11 ^a,b,e^	0.63 ± 0.23 ^a,b^	0.5 ± 0.00 ^a^	0.67 ± 0.3 ^a^
14	MIC	0.77 ± 0.11 ^a,b,h^	0.41 ± 0.11 ^a^	0.33 ± 0.3 ^a,b,d^	0.35 ± 0.00 ^a,f,c^	0.40 ± 0.1 ^a,f^	0.80 ± 0.17 ^a,e^	0.20 ± 0.04 ^a,b,d,e^	0.42 ± 0.11 ^a,b^
	MBC	1.27 ± 0.23 ^a,b^	0.7 ± 0.26 ^a,f^	0.7 ± 0.00 ^a,b^	0.77 ± 0.11 ^a,b^	0.7 ± 0.26 ^a,c^	1.13 ± 0.11 ^a,b,d^	0.42 ± 0.11 ^a,d^	0.7 ± 0.00 ^a^
15	MIC	1.07 ± 0.06 ^a,b,d^	0.70 ± 0.26 ^a,c^	0.28 ± 0.11 ^a,b,d^	1.13 ± 0.06 ^a,b,d^	0.70 ± 0.25 ^a^	2.13 ± 0.11 ^a^	0.30 ± 0.4 ^a,b,e,g^	0.28 ± 0.014 ^a^
	MBC	2.13 ± 0.11 ^a,b,f^	1.23 ± 0.23 ^a,b^	0.7 ± 0.3 ^a,b,c,f^	0.63 ± 0.14 ^a,b,e^	1.1 ± 0.00 ^a^	4.27 ± 0.23 ^a^	0.70 ± 0.26 ^a,b^	0.57 ± 0.03 ^a,c^
16	MIC	0.9 ± 0.1 ^a,b^	0.43 ± 0.06 ^a^	0.47 ± 0.11 ^a,b,f^	0.4 ± 0.00 ^a,i^	0.3 ± 0.17 ^a,b,e^	0.43 ± 0.06 ^a,f,e^	0.47 ± 0.11 ^a^	0.4 ± 0.00 ^a,b^
	MBC	1.63 ± 0.11 ^a^	0.87 ± 0.11 ^a,b,c,d^	0.8 ± 0.09 ^a,b,d^	0.73 ± 0.11 ^a,b,c,j^	0.83 ± 0.06 ^a,b,c^	0.8 ± 0.00 ^a,c^	0.87 ± 0.11 ^a,b,f^	0.70 ± 0.17 ^a^
17	MIC	0.83 ± 0.14 ^a,b,h^	0.23 ± 0.06 ^a^	0.40 ± 0.04 ^a,e,f^	0.21 ± 0.03 ^a,b^	0.19 ± 0.01 ^a,d^	0.18 ± 0.02 ^a,c^	0.23 ± 0.06 ^a^	0.40 ± 0.04 ^a,b^
	MBC	1.33 ± 0.3 ^a,b^	0.45 ± 0.12 ^a,c^	0.80 ± 0.09 ^a,c,d^	0.34 ± 0.07 ^a,b^	0.38 ± 0.00 ^a,g^	0.40 ± 0.04 ^a,d^	0.42 ± 0.07 ^a,d^	0.80 ± 0.09 ^a,b,e^
18	MIC	1.13 ± 0.06 ^a,b,k^	0.70 ± 0.2 ^a,c,b,e,g^	0.67 ± 0.2 ^a,b.d^	0.70 ± 0.3 ^a,b,c,d,f^	0.60 ± 0.1 ^a^	1.1 ± 0.06 ^a,b,e^	0.70 ± 0.3 ^a,b,e,g^	0.55 ± 0.00 ^a,k^
	MBC	2.13 ± 0.11 ^a,b^	1.07 ± 0.06 ^a,b^	1.23 ± 0.23 ^a,b,d^	1.17 ± 0.11 ^a,b,j^	1.07 ± 0.06 ^a^	2.30 ± 0.17 ^a,b^	1.23 ± 0.23 ^a,b^	1.03 ± 0.11 ^a^
19	MIC	0.67 ± 0.30 ^a,l,j^	0.33 ± 0.14 ^a^	0.25 ± 0.00 ^a^	0.28 ± 0.06 ^a,b,f^	0.32 ± 0.11 ^a,b,c^	0.5 ± 0.00 ^a,b,f^	0.27 ± 0.11 ^a^	0.37 ± 0.2 ^a,e^
	MBC	1.17 ± 0.30 ^a,b,f,i^	0.67 ± 0.3 ^a,f^	0.5 ± 0.00 ^a,b^	0.57 ± 0.11 ^a,b^	0.60 ± 0.17 ^a,b^	1.16 ± 0.28 ^a,c^	0.67 ± 0.28 ^a,b,f^	0.43 ± 0.11 ^a,e^
20	MIC	0.08 ± 0.01 ^a,b^	0.075 ± 0.00 ^a,g^	0.075 ± 0.00 ^a,b,f^	0.07 ± 0.01 ^a^	0.17 ± 0.03 ^a,b^	0.20 ± 0.08 ^a,e^	0.15 ± 0.00 ^a,b^	0.13 ± 0.03 ^a^
	MBC	0.17 ± 0.03 ^a,b^	0.20 ± 0.08 ^a,b,d^	0.15 ± 0.00 ^a,b^	0.17 ± 0.03 ^a,b^	0.33 ± 0.06 ^a,c,e^	0.3 ± 0.00 ^a,b,c^	0.37 ± 0.11 ^a,c,f^	0.27 ± 0.06 ^a,b,e^
21	MIC	0.2 ± 0.00 ^a,b,j,k^	0.13 ± 0.06 ^a,e^	0.27 ± 0.11 ^a^	0.1 ± 0.00 ^a,b^	0.17 ± 0.11 ^a,b^	0.20 ± 0.17 ^a,e^	0.13 ± 0.06 ^a,b^	0.27 ± 0.11 ^a,g^
	MBC	0.4 ± 0.00 ^a,b,d^	0.17 ± 0.06 ^a,b,f^	0.47 ± 0.11 ^a,b^	0.2 ± 0.00 ^a,b,c^	0.27 ± 0.11 ^a,b,c^	0.33 ± 0.23 ^a,b^	0.30 ± 0.17 ^a,b,c^	0.50 ± 0.17 ^a^
22	MIC	0.27 ± 0.1 ^a,b,d,h^	0.1 ± 0.00 ^a,c^	0.13 ± 0.06 ^a^	0.17 ± 0.11 ^a,d^	0.13 ± 0.1 ^a^	0.27 ± 0.11 ^a,b^	0.23 ± 0.06 ^a,e^	0.1 ± 0.00 ^a,b^
	MBC	0.33 ± 0.11 ^a,b^	0.27 ± 0.11 ^a,b,d^	0.30 ± 0.17 ^a,b^	0.2 ± 0.00 ^a,b,c^	0.17 ± 0.06 ^a,b,c^	0.47 ± 0.11 ^a,b^	0.50 ± 0.17 ^a^	0.2 ± 0.00 ^a,b,e^
23	MIC	0.8 ± 0.00 ^a,b,d,h,k^	0.33 ± 0.11 ^a,c^	0.73 ± 0.11 ^a,b,e^	0.77 ± 0.06 ^a,b,c,d,f^	0.33 ± 0.11 ^a,b,e^	0.8 ± 0.00 ^a,e^	0.27 ± 0.11 ^a^	0.37 ± 0.06 ^a^
	MBC	1.53 ± 0.11 ^a,b,f^	0.87 ± 0.11 ^a,b,c^	1.53 ± 0.11 ^a,d^	1.40 ± 0.34 ^a,b,j^	0.87 ± 0.11 ^a,b,f,e^	1.56 ± 0.06 ^a,b,c^	0.4 ± 0.00 ^a,f^	0.87 ± 0.11 ^a,b,e^
24	MIC	0.87 ± 0.11 ^a,b,k^	0.47 ± 0.11 ^a^	0.43 ± 0.06 ^a^	0.4 ± 0.00 ^a,i^	0.33 ± 0.11 ^a,b^	0.73 ± 0.11 ^a^	0.27 ± 0.11 ^a^	0.23 ± 0.06 ^a,c^
	MBC	1.40 ± 0.35 ^a,b,f^	0.87 ± 0.11 ^a,b,d^	0.73 ± 0.11 ^a,b,d^	0.8 ± 0.00 ^a,b^	0.87 ± 0.11 ^a,b,f,e^	1.6 ± 0.00 ^a,b,c,d^	0.53 ± 0.23 ^a,b^	0.47 ± 0.11 ^a^
25	MIC	0.18 ± 0.06 ^a,b,j^	0.08 ± 0.01 ^a,b,g^	0.08 ± 0.01 ^a,f^	0.075 ± 0.00 ^a,b,c^	0.075 ± 0.00 ^a,b,f^	0.07 ± 0.01 ^a,b,c,f^	0.075 ± 0.00 ^a^	0.08 ± 0.01 ^a,b^
	MBC	0.3 ± 0.00 ^a,i^	0.18 ± 0.06 ^a,c^	0.13 ± 0.03 ^a,b^	0.15 ± 0.00 ^a,b^	0.20 ± 0.08 ^a,b,c^	0.17 ± 0.03 ^a,b,c^	0.15 ± 0.00 ^a,b^	0.13 ± 0.03 ^a,b,e^
26	MIC	0.67 ± 0.3 ^a,b,k^	0.33 ± 0.14 ^a,c^	0.27 ± 0.03 ^a^	0.25 ± 0.00 ^a,c^	0.32 ± 0.11 ^a,b,c^	0.67 ± 0.28 ^a,b^	0.25 ± 0.00 ^a,g^	0.33 ± 0.14 ^a^
	MBC	1.17 ± 0.3 ^a,b,d^	0.67 ± 0.3^a,f^	0.47 ± 0.06^a,b^	0.57 ± 0.11^a,b^	0.67 ± 0.28 ^a,b,e^	1 ± 0.00^a,b,d^	0.5 ± 0.00^a^	0.67 ± 0.3^a^
27	MIC	1.33 ± 0.3 ^a,d,h,k^	0.33 ± 0.11 ^a,c^	0.37 ± 0.06 ^a,b,c,d^	0.43 ± 0.06 ^a,d,f^	0.4 ± 0.00 ^a,f^	0.47 ± 0.11 ^a,c^	0.50 ± 0.17 ^a^	0.37 ± 0.06 ^a^
	MBC	2.67 ± 0.6 ^a,b^	0.87 ± 0.11 ^a,b,f^	0.77 ± 0.06 ^a^	0.70 ± 0.17 ^a,e,j^	0.73 ± 0.11 ^a,c^	0.87 ± 0.11 ^a,b,e^	0.8 ± 0.00 ^a,b,c^	0.87 ± 0.11 ^a,b,e^
28	MIC	0.5 ± 0.00 ^a,b^	0.16 ± 0.03 ^a^	0.14 ± 0.01 ^a,b,f^	0.16 ± 0.03 ^a,b,c^	0.13 ± 0.03 ^a,d^	0.14 ± 0.00 ^a,b,f^	0.17 ± 0.06 ^a,b,c^	0.15 ± 0.02 ^a,e^
	MBC	1 ± 0.00 ^a,b,i^	0.28 ± 0.01 ^a^	0.30 ± 0.03 ^a,b,d^	0.35 ± 0.13 ^a,b^	0.25 ± 0.05 ^a,b^	0.28 ± 0.00 ^a,b,e^	0.29 ± 0.11 ^a,b^	0.27 ± 0.017 ^a,b,e^
Streptomycin	MIC	0.10 ± 0.04 ^a,d^	0.10 ± 0.03 ^a,b^	0.025 ± 0.02 ^a,b,e^	0.15 ± 0.04 ^a,f^	0.10 ± 0.05 ^a,e^	0.05 ± 0.06 ^a^	0.025 ± 0.01 ^a^	0.10 ± 0.05 ^a^
	MBC	0.20 ± 0.01 ^a^	0.20 ± 0.06 ^a,b^	0.05 ± 0.01 ^a,b^	0.30 ± 0.02 ^a,b^	0.20 ± 0.01 ^a,b^	0.1 ± 0.00 ^a,b^	0.05 ± 0.02 ^a,b^	0.20 ± 0.02 ^a,b^
Ampicillin	MIC	0.15 ± 0.02 ^a,d^	0.10 ± 0.03 ^a,b^	0.10 ± 0.05 ^a,b,e^	0.15 ± 0.03 ^a,d,f,i^	0.30 ± 0.03 ^a,e^	0.10 ± 0.04 ^a^	0.10 ± 0.04 ^a^	0.10 ± 0.05 ^a^
	MBC	0.20 ± 0.01 ^a^	0.20 ± 0.06 ^a,b^	0.15 ± 0. 04 ^a,b^	0.30 ± 0.01 ^a^	0.50 ± 0.02 ^a,b^	0.15 ± 0.01 ^a,b^	0.15 ± 0.03 ^a,b^	0.15 ± 0.01 ^a,b^

^a^^–l^ Values with different lowercase letters in the same column are significantly different (*p*-value ≤ 0.05)

**Table 6:** Sample-set of wine lees and their general characteristics.

A/A	Variety	Variety Code	Color ^1^	Origin ^2^	Fermentation Stage ^3^
1	Kidonitsa	1	1	2	1
2	Kidonitsa	1	1	2	2
3	Savvatiano	2	1	1	2
4	Savvatiano	2	1	1	2
5	Savvatiano	2	1	1	1
6	Savvatiano	2	1	1	1
7	Savvatiano	2	1	1	2
8	Savvatiano	2	1	1	2
9	Chardonnay	3	1	1	2
10	Chardonnay	3	1	1	2
11	Chardonnay	3	1	2	1
12	Moschofilero	4	1	2	2
13	Moschofilero	4	1	2	2
14	Moschofilero	4	1	2	2
15	Moschofilero	4	1	2	2
16	Grenache rouge 1	5	2	1	1
17	Grenache rouge 2	5	2	1	1
18	Merlot	6	2	1	1
19	Merlot	6	2	1	1
20	Merlot	6	2	2	2
21	Cabernet	7	2	2	2
22	Cabernet	7	2	2	2
23	Agiorgitiko	8	2	2	2
24	Agiorgitiko	8	2	2	1
25	Agiorgitiko	8	2	2	2
26	Agiorgitiko	8	2	2	2
27	Agiorgitiko	8	2	2	2
28	Agiorgitiko	8	2	2	2

^1^ Color: 1 = White, 2 = Red; ^2^ Origin: 1 = Attica, 2 = Peloponnese; ^3^ Fermentation stage: 1 = Pre-fermented, 2 = Post-fermented.

## Data Availability

The data presented in this study are available in Supplementary data.

## Supplementary Materials

The following are available online at https://www.mdpi.com/article/10.3390/molecules27072189/s1, Figure S1: Contour plots of: (a) extraction time vs MW power; (b) solvent/material ratio vs MW power; (c) solvent/material ratio vs extraction time, Table S1: randomized experimental runs and (poly)phenols extraction yield of 2^3^ and BBD models, Table S2: ANOVA table of: (a) 2^3^ design; (b) BBD model for MAE of wine lees (poly)phenols, Table S3: predicted and observed extraction yields of wine lees at optimal experimental combinations proposed by the BBD model, Table S4: organic acid and sugar content of the sediment extracts of wine lees expressed as mg g^−1^ of dry sample, Table S5: antibacterial activity of wine lees sediment against ATCC bacteria (mg mL^−1^), Table S6: moisture % (*w*/*w*) of the sediment of wine lees samples.

## Sample Availability

Samples of the compounds are not available from the authors.
