# Supplementary material for: Towards the Optimization of Microwave-Assisted Extraction and the Assessment of Chemical Profile, Antioxidant and Antimicrobial Activity of Wine Lees Extracts
Source: Molecules. 2022 Mar 28;27(7):2189. doi: 10.3390/molecules27072189 (PMC9000764; doi:10.3390/molecules27072189)
Supplement: Supplementary file 1 [file molecules-27-02189-s001.zip › molecules-1646027-supplementary.pdf]

molecules

MDPI

Article

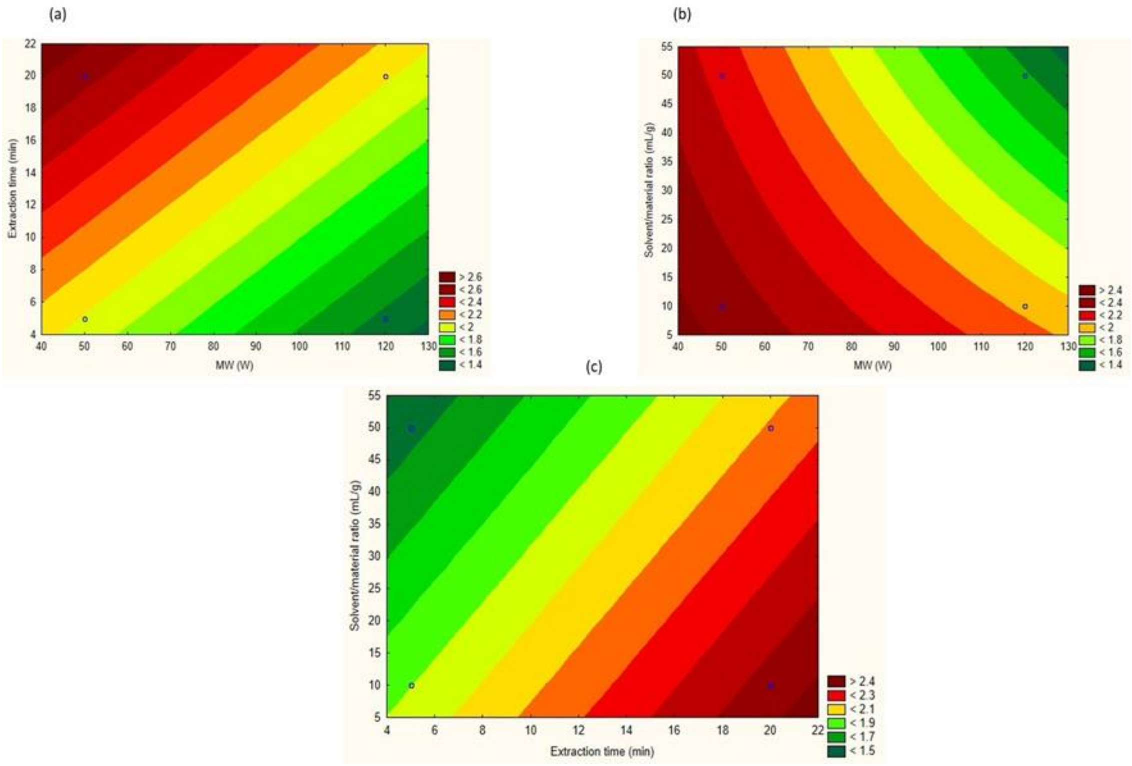

Figure S1. Contour plots of: **(a)** extraction time vs MW power; **(b)** solvent/material ratio vs MW power; **(c)** solvent/material ratio vs extraction time.

Table S1. Randomized experimental runs and (poly)phenols extraction yield of 2^3 and BBD models.

Standard run	Coded combinations (x_1, x_2, x_3)	Extraction yield (mg of GAE g^{-1} dry sediment) (±stdev), n=3 [1]
	2^3 design	
**2**	-1,-1,+1	1.92(±0.21) [b]
**3**	-1,+1,-1	1.99(±0.86) [b]
**6**	+1,-1,+1	1.27(±0.57) [b]
**4**	-1,+1,+1	2.440(±0.074) [a, b]
**5**	+1,-1,-1	1.99(±0.26) [b]
**8**	+1,+1,+1	1.36(±0.22) [b]
**1**	-1,-1,-1	1.76(±0.18) [b]
**7**	+1,+1,-1	3.31(±0.56) [a]
	BBD model	
**16**	0,0,0	3.33(±0.31) [b, c, d]
**4**	-1,+1,0	3.77(±0.17) [a]
**12**	0,+1,+1	3.48(±0.10) [a, b, c]
**3**	-1,+1,0	3.72(±0.11)
**13**	0,0,0	3.78(±0.17) [a]
**6**	+1,0,-1	3.802(±0.034) [a]
**11**	0,-1,+1	3.472(±0.049) [a, b, c]
**8**	+1,0,+1	3.279(±0.021) [c, d]

[1] n=the number of replicates; [a-e:] values with different lowercase letters are significantly different (p-value≤0.05).

Table S2. ANOVA table of: **(a)** 2^3 design; **(b)** BBD model for MAE of wine lees (poly)phenols.

(a)

**Equation terms**	**Sum of squares (SS)**	***F*-value**	***p*-value**
x_1	0.535	4.67	0.097
x_2	0.588	5.13	0.086
x_1x_3	1.34	11.66	0.027 [1]
**Pure error (degrees of freedom)**	0.458 (4)		
**Total SS (degrees of freedom)**	2.92 (7)		

[1] significant equation terms (*p*-value≤0.05)

(b)

**Equation terms**	**Sum of squares (SS)**	***F*-value**	***p*-value**
x_1	0.030	1.64	0.24
x_1^2	0.083	4.54	0.071
x_2	0.323	17.68	0.0040 [1]
x_1x_2	0.102	5.61	0.050 [1]
$x_1x_2^2$	0.090	4.90	0.062
$x_1^2x_2$	0.119	6.49	0.038 [1]
x_1x_3	0.334	18.24	0.037 [1]
$x_1^2x_3$	0.057	3.10	0.12
**Pure error (degrees of freedom)**	0.128 (7)		
**Total SS (degrees of freedom)**	1.15 (15)		

[1] significant equation terms (*p*-value≤0.05)

Table S3. Predicted and observed extraction yields of wine lees at optimal experimental combinations proposed by the BBD model.

MAE	MW power (W)	Extraction time (min)	Solvent/material ratio (mL g^{-1})	Predicted extraction yield (mg of GAE g^{-1} dry sample)	Experimental extraction yield (mg of GAE g^{-1} dry sample) (±stdev), n=3
**Run A**	44	25	60	3.48	3.39(±0.10) [a]
**Run B**	50	30	60	3.67	3.541(±0.082) [a]
**Run C**	54	35	60	3.77	3.58(±0.17) [1 a]

Table S4. Moisture % (w/w) of the sediment of wine lees samples.

A/A	Variety	% Moisture of sediment (w/w)
1	Kidonitsa	65.2
2	Kidonitsa	81.5
3	Savatiano	76.1
4	Savatiano	76.5
5	Savatiano	73.4
6	Savatiano	80.3
7	Savatiano	57.9
8	Savatiano	41.8
9	Chardonnay	61.0
10	Chardonnay	72.4
11	Chardonnay	61.5
12	Moschofilero	58.8
13	Moschofilero	60.0
14	Moschofilero	64.9
15	Moschofilero	76.9
16	Grenache rouge 1	79.8
17	Grenache rouge 2	76.8
18	Merlot	77.0
19	Merlot	67.2
20	Merlot	62.6
21	Cabernet	70.3
22	Cabernet	70.8
23	Agiorgitiko	44.7
24	Agiorgitiko	49.8
25	Agiorgitiko	67.1
26	Agiorgitiko	64.0
27	Agiorgitiko	80.2
28	Agiorgitiko	74.9

Table S5. Real and coded values of MAE extraction factors.

Coded values	-1	0	+1
		2^3 design	
MW power (X_1, W)	50	-	120
Extraction time (X_2, min)	5	-	20
Solvent/material ratio (X_3, mL g^{-1})	10	-	50
		BBD	
MW power (X_1, W)	40	50	60
Extraction time (X_2, min)	20	25	30
Solvent/material ratio (X_3, mL g^{-1})	45	50	55

1. Isolation and Determination of Clinical Bacteria

Methicillin-Resistant Staphylococcus aureus (MRSA)

This strain is isolated from cows with subclinical mastitis. Milk samples were streaked onto Columbia agar plates (Torlak, Serbia) containing 5% sheep blood, Baird–Parker agar plates (HiMedia, India) and chromogenic culture media (chromID MRSA, bioMerieux). After incubation at 37°C for 24 h, the colonies were presumptively identified according to morphological features, pigment production, Gram stain results, catalase and oxidase test results, type of haemolysis and characteristic growth on BP agar and chromID MRSA.

Colonies (suspicious) to *S. aureus* on the blood agar and green colonies on chromogenic media were transferred to individual plates to obtain pure culture. The identification was confirmed using BBL Crystal, G/P, ID kit (Becton Dickinson). Antimicrobial susceptibility testing was performed by disk diffusion method with cefoxitin discs 30 μg (Rosco, Denmark) in accordance to the Clinical and Laboratory Standard Institute recommendations. All isolated strains of S. aureus were tested for the presence of penicillin-binding protein (PBP2) with latex agglutination test (Slidex MRSA Detection, bioMerieux). *Staphylococcus aureus* ATCC 25923 was used as the control strain. All isolates were tested for the presence of mecA gene by PCR [54].

Escherichia coli

Samples of rectal swabs, feces and intestines from diseased pigs were taken. In order to isolate *E. coli* strains the following nutrition media were used: MacConkey agar (Torlak), Columbia agar (Torlak) with 5% defibrinated sheep blood and Brilliant Green agar (Torlak). For the identification of the isolated strains, laboratory tests with the following nutritious media and reagents were used: Simmons citrate agar (Torlak), MR/VP broth (Torlak), Christensen urea agar (Torlak), peptone water for indole test (Torlak), catalase and oxidase, triple sugar agar (Torlak), as well as identification systems BBL Crystal Entero/nonfermenter ID kit (Becton Dickinson). Sensitivity studies on the isolated bacteria were completed by the disc diffusion method on Mueller–Hinton agar with the use of antibiogram discs (Bioanalyse) and tablets (Torlak) for the following antibiotics: penicillin, ampicillin, amoxicillin, tetracycline, neomicin,

gentamicin, colistin, ceftriaxon, sulphamethaxasole with trimetoprim, enrofloxacin and florfenicol. All isolated *E. coli* strains were resistant to all tested antibiotics with the exception of enrofloxacin, colistin and florfenicol [55].

Pseudomonas aeruginosa

The strains were isolated from cats and dogs. Samples were inoculated on Columbia agar plates (Torlak, Serbia) containing 5% sheep blood, nutrition agar (HiMedia) and MacConkey agar (Torlak) and incubated in aerobic conditions at a temperature of 37 °C and 42 °C for 24 h. Pure cultures were identified on morphological and biochemical characteristics. For identification of pigment production sub-cultivation on corresponding media was performed. The identification was confirmed using BBL Crystal, Entero/nonfermenter, ID kit (Becton Dickinson). Sensitivity studies were completed by the disc diffusion method on Mueller–Hinton agar with the use of antibiogram discs (Bioanalyse) and tablets (Torlak) for the following antibiotics: penicillin, ampicillin, amoxicillin, tetracycline, neomicin, gentamicin, ceftriaxon, sulphamethaxasole with trimetoprim, enrofloxacin and florfenicol. All isolated *Pseudomonas aeruginosa* strains were resistant to all tested antibiotics with the exception of enrofloxacin and florfenicol [55].

54. Stegger, M.; Andersen, P.S.; Kearns, A.; Pichon, B.; Holmes, M.A.; Edwards, G.; Laurent, F.; Teale, C.; Skov, R.; Larsen, A.R. Rapid Detection, Differentiation and Typing of Methicillin-Resistant Staphylococcus Aureus Harbouring Either MecA or the New MecA Homologue MecALGA251. *Clinical Microbiology and Infection* **2012**, *18*, 395–400, doi:10.1111/j.1469-0691.2011.03715.x.
55. Wilker, M. Methods for Dilution Antimicrobial Susceptibility Tests for Bacteria That Grow Aerobically : Approved Standard. *CLSI (NCCLS)* **2006**, *26*, M7-A7.
